# Urinary epidermal growth factor, monocyte chemoattractant protein-1 or their ratio as predictors for rapid loss of renal function in type 2 diabetic patients with diabetic kidney disease

**DOI:** 10.1186/s12882-018-1043-x

**Published:** 2018-09-21

**Authors:** Bancha Satirapoj, Rattanawan Dispan, Piyanuch Radinahamed, Chagriya Kitiyakara

**Affiliations:** 1 Division of Nephrology, Department of Medicine, Phramongkutklao Hospital and College of Medicine, Bangkok, Thailand; 2 Division of Nephrology, Department of Medicine, Faculty of Medicine Ramathibodi Hospital, Mahidol University, 270 Rama 6 Rd, Bangkok, 10400 Thailand

**Keywords:** Biomarker, Diabetic nephropathy, Monocyte chemoattractant protein-1 (MCP-1), Epidermal growth factor (EGF); kidney; cytokine

## Abstract

**Background:**

Increased monocyte chemoattractant protein-1 (MCP-1) and decreased epidermal growth factor (EGF) are promising biomarkers to predict progressive decline in kidney function in non-diabetic kidney diseases. We aimed to evaluate the performance of urinary EGF, MCP-1 or their ratio in predicting rapid decline of GFR in a cohort of Type 2 diabetic patients (T2DM) with diabetic kidney disease (DKD).

**Methods:**

T2DM patients (*n* = 83) with DKD at high risk for renal progression were followed up prospectively. The baseline urine values of MCP-1 to creatinine ratio (UMCP-1), EGF to creatinine ratio (UEGF), EGF to MCP-1 ratio (UEGF/MCP-1) and albumin to creatinine ratio (UACR) were measured. The primary outcome was a decline in estimated glomerular filtration rate (GFR) of ≥25% yearly from baseline.

**Results:**

During follow-up time of 23 months, patients with rapid decline in estimated GFR of ≥25% yearly from baseline had significantly higher baseline levels of UMCP-1, and UACR and lower UEGF and UEGF/MCP-1 ratio. All renal biomarkers predicted primary outcomes with ROC (95%CI) for UMCP-1=0.73 (0.62-0.84), UEGF=0.68 (0.57-0.80), UEGF/MCP-1=0.74 (0.63-0.85), and UACR =0.84 (0.75-0.93). By univariate analysis, blood pressure, GFR, UACR, UMCP-1, UEGF, and UEGF/MCP-1 were associated with rapid decline GFR. By multivariate analysis, UACR, systolic blood pressure, and UMCP-1 or UEGF/MCP-1 were independently associated with rapid GFR decline.

**Conclusions:**

UMCP-1 or UEGF/MCP-1 ratio were associated with rapid renal progression independent from conventional risk factors in DKD.

**Electronic supplementary material:**

The online version of this article (10.1186/s12882-018-1043-x) contains supplementary material, which is available to authorized users.

## Background

Diabetic kidney disease (DKD) is a major complication of type 2 diabetes mellitus (T2DM) with up to 40% of DKD patients progressing to end stage renal disease (ESRD) [1]. Despite the improvements in the treatment for DKD, the risk of ESRD remains high. Potential new therapy must be evaluated in clinical trials, which are very costly. A successful trial must accumulate enough end-points to give adequate power for detecting a risk reduction between the placebo and the treated group. Biomarkers that can identify and target high risk patients for clinical studies or more aggressive therapy would be extremely useful. The paradigm of the natural history of DKD continues to evolve. DKD clearly does not follow the pattern of glomerular hyperfiltration progressing from microalbuminuria to increasing degrees of overt albuminuria and declining GFR in many patients [2]. Macrovascular disease rather than classical diabetic nephropathy has been increasingly recognized in the pathogenesis of GFR decline in many DKD patients [3] [4]. Although, albuminuria is an important marker to diagnose and predict the progression of DKD [2], nearly half of T2DM patients may have decreased glomerular filtration rate (GFR) before the onset of albuminuria. Conventional biomarkers cannot accurately identify T2DM patients at higher risk of rapid GFR decline and new biologic markers that can predict DKD progression are required.

DKD has been viewed traditionally as a disease of accelerated matrix deposition leading to progressive glomerular and tubulointerstitial fibrosis with the final consequence of ESRD [2]. More recently, an important role of inflammatory cells such as monocytes/macrophages has been recognized in the pathogenesis of DKD progression [5]. Monocyte chemoattractant protein-1 (MCP-1), a member of the CC chemokine family promotes macrophage accumulation both in animal models and several renal diseases [6–8]. Increased urinary MCP-1 levels have been shown to predict adverse outcomes in proliferative kidney diseases such as lupus nephritis [9]. Previous investigations have shown that the renal MCP-1 expression is also elevated in DKD. Urinary MCP-1 levels correlated with the degree of tubulointerstitial leucocyte infiltration [10, 11], but at present, its role in predicting renal prognosis in DKD remains unclear.

The balance between protective growth factors and pro-inflammatory cytokines likely determines the degree of renal tissue damage and disease progression in DKD. In contrast to MCP-1, epidermal growth factor (EGF), a peptide growth factor probably has a protective role during kidney injury. EGF expression within the kidney is decreased in several kidney diseases [12, 13]. Low urinary EGF levels have been found to be predictive of kidney function decline in non-diabetic renal diseases. Therefore, both urinary MCP-1 and EGF could serve as favorable biomarkers for kidney damage in DKD. Previously, it has been shown that urinary EGF/MCP-1 ratio was a better prognosticator of long term outcome compared to either cytokine alone in IgA nephropathy [14]. Currently, few studies have evaluated the roles of MCP-1 or EGF in predicting DKD progression across a broad spectrum of kidney function and none have evaluated the role of their ratio. This study aimed to test the hypothesis that baseline levels of urinary MCP-1 and EGF or their ratio would predict rapid decline of estimated GFR in a cohort of T2DM patients with CKD independent of conventional clinical risk factors. We also evaluated the biomarkers in subgroups with high cardiovascular risk and those low albuminuria to explore the potential value of these biomakers in subjects who may have non-classical mechanisms for DKD progression.

## Methods

### Study design

This prospective cohort study is comprised of T2DM subjects with DKD at risk for renal progression. The subjects were recruited in 2014 to 2016 and followed for at least 12 months at the outpatient clinic, Department of Internal Medicine, Phramongkutklao Hospital. The study was conducted according to the Declaration of Helsinki, and approved by the Ethics Committee of the Faculty of Medicine, Ramathibodi Hospital and the Royal Thai Army Medical Department. All the subjects gave written informed consent. Inclusion criteria were: age ≥ 18 years, T2DM with predialysis CKD stage 1 to 5 (persistent micro or macroalbuminuria or estimated GFR < 60 mL/min/1.73 m^2^ not on dialysis) and a duration of diabetes of at least 5 years or uncontrolled blood pressure (BP) for 6 months (> 150/90 mmHg). Exclusion criteria included urinary tract infection, ESRD, acute kidney injury, as defined by a rapid rise in serum creatinine and/or fall in urine output according to KDIGO classification 3 months prior to inclusion or during follow-up [15], pregnancy, T1DM and patient life expectancy < 1 year.

Patient history was recorded by interview and confirmed by assessment of medical records and drug prescriptions. Demographic and anthropometric parameters were recorded. Blood pressure (BP) was measured using a standard mercury sphygmomanometer. An average of three measurement values was used. After the baseline assessments, patients were followed prospectively until the end of the observation period. To avoid loss during follow-up, patients were personally contacted if they missed any appointments and at the end of the study.

Patients were also classified according to cardiovascular risks. Patients with prior myocardial infarction, angina or stroke were classified as having a ‘history of cardiovascular events’. Patients with a ‘high cardiovascular disease’ was defined as a 10-year cardiovascular risk ≥10% by Framingham Coronary Heart Disease Risk Score [16].

### Laboratory measurements

Blood and urine samples were taken in the morning before any food intake. Common biochemical parameters including urea, creatinine, hemoglobin A1C, serum lipids and electrolytes, albumin, hemoglobin and proteinuria were measured at baseline in all patients, according to standard methods in a routine clinical laboratory. Estimated GFR (eGFR) was calculated using the Chronic Kidney Disease Epidemiology Collaboration (CKD-EPI) equation [17]. Urine albumin was measured on a nephelometric analyzer and urine creatinine was measured on a multiple analyzer (Modular P Chemistry Analyzer; Roche Diagnostics). Urine albumin and creatinine for urine samples collected from participants and albuminuria was reported as UACR.

### Urine MCP-1 and EGF assay

Urine samples were collected at baseline. Thirty milliliters of fresh urine was centrifuged at 4000 rpm for 10 min, then stored at − 80 °C until assayed. Sandwich ELISA kits (Quantikine ELISA Immunoassay R&D systems, Minneapolis, USA) was used to quantitate urinary MCP-1 and EGF levels using the manufacturer’s instructions. Duplicate samples were measured by a researcher who did not have information on the clinical data using TECAN Infinite M200 Pro microplate reader. The results were calculated using Magellan Tracker software (Tecan Group Ltd., Mannedorf, Switzerland). Urine MCP-1 and EGF levels were expressed as ng per mg of creatinine (UMCP-1 and UEGF). Ratio of Urine EGF and MCP-1 were also calculated. The intra- assay coefficients of variation were 3.2% for EGF and 2.6% for MCP-1.

### Renal outcome

The primary outcome was *Rapid GFR decline*, defined as decreased estimated GFR decline ≥ 25% per year. This was determined for each follow-up time point by calculating the percent GFR change from baseline for time points within 1 year of urine collection or from the preceding 12 months for time-points beyond one year.

### Statistical analyses

Normally distributed variables are shown as mean ± standard deviation and compared using the Pearson correlation or unpaired t-test. Variables that are Non-normally distributed are presented as median [25th, 75th percentile] and compared by the Spearman coefficients or the Mann-Whitney U test. Categorical variables are summarized as percentages and compared using the chi-square test. Receiver-operating characteristic (ROC) curves were generated and the area under the curves (AUC) were calculated to evaluate the sensitivity and specificity for UMCP-1, UEGF, UEGF/MCP-1 ratio, and UACR for *Rapid GFR decline*. The Youden index was used to determine the optimal cut-off value [18]. To evaluate the prognostic value of each biomarker to predict *Rapid GFR decline*, event-free survival times were compared between patients with high and low biomarker levels. Time to event was the period to *Rapid GFR decline*. Patients were censored if they reached November 30th, 2017 without events. Survival functions were assessed with Kaplan-Meier. Hazard ratios for outcomes associated with *Rapid GFR decline* were determined by Cox proportional hazards for each biomarker and conventional risk factors using three models separately for UMCP-1, UEGF or UEGF/MCP-1. In addition to the analysis of all subjects, we also performed subgroup analysis of subjects with high cardiovascular risk or those low albuminuria. A *p* value of < 0.05 was considered statistically significant.

## Results

### Baseline characteristics

The baseline characteristics of the study cohort (*n* = 83) are shown in Table 1. The mean age was 66.6 ± 9.8 years, and more than half were male (63.8%). Duration of T2DM was 13.9 ± 8.9 years and all patients had follow-up time of 24 [11, 19] months. Mean estimated GFR was 45.0 ± 28.4 mL/min/1.73 m^2^, UACR was 283.69 [34.47, 762.85] mg/gCr and HbA1c was 7.4 ± 1.6%. Seventy-nine patients (96.3%) were hypertensive, and 57 (68.7%) had GFR < 60 mL/min/1.73 m^2^. The prevalence of patients with prior cardiovascular events was 3.7% and 44.6% were considered to have high cardiovascular risk.

**Table 1:** Baseline characteristics and laboratory data

Parameters	All patients (*N* = 83)	GFR decline < 25% per year (*N* = 46)	GFR decline ≥ 25% per year (*N* = 37)	*P*-value
Age (year)	66.6 ± 9.8	67.1 ± 8.9	65.9 ± 10.9	0.595
Male (%)	53 (63.9%)	29 (63.0%)	24 (64.9%)	0.864
Duration of DM (years)	13.9 ± 8.9	14.8 ± 8.3	13.2 ± 9.6	0.463
CKD staging				
CKD I	7 (8.4%)	4 (8.7%)	3 (8.1%)	0.065
CKD II	19 (22.9%)	14 (30.4%)	5 (13.5%)	
CKD III	26 (31.3%)	17 (36.9%)	9 (24.3%)	
CKD IV	16 (19.3%)	5 (10.9%)	11 (29.7%)	
CKD V	15 (18.1%)	6 (13.0%)	9 (24.3%)	
Comorbid diseases				
Hypertension (%)	79 (96.3%)	44 (95.7%)	35 (94.6%)	0.586
Dyslipidemia (%)	72 (87.8%)	43 (95.6%)	29 (78.4%)	0.037
History of cardiovascular events (%)	3 (3.7%)	2 (4.4%)	1 (2.7%)	1.000
Framingham risk score	15.63 ± 10.29	13.83 ± 8.07	17.76 ± 12.22	0.152
High cardiovascular risk^a^ (%)	37 (44.6%)	21 (45.7%)	16 (43.2%)	0.605
Anemia (%)	51 (61.5%)	22 (47.8%)	29 (78.4%)	0.004
Medications				
RAAS blockers (%)	45 (54.2%)	27 (58.7%)	18 (48.7%)	0.361
Insulin (%)	29 (35.4%)	14 (31.1%)	15 (40.5%)	0.374
ASA (%)	50 (60.9%)	28 (62.2%)	22 (59.5%)	0.799
Clinical parameters				
SBP (mmHg)	143.1 ± 22.4	135.5 ± 16.3	152.4 ± 25.4	0.001
DBP (mmHg)	77.7 ± 13.9	74.8 ± 12.2	81.2 ± 15.2	0.037
BMI (kg/m^2^)	27.5 ± 5.0	27.3 ± 5.2	27.73 ± 4.9	0.736
Laboratory parameters				
GFR (mL/min/1.73m^2^)	45.0 ± 28.4	51.9 ± 27.6	36.4 ± 27.4	0.012
Median UACR (mg/g creatinine)	283.7 [34.5, 762.9]	49.7 [19.9, 261.3]	673.4 [412.6, 2627.6]	< 0.001
FPG (mg/dL)	150.6 ± 74.7	146.4 ± 78.9	155.8 ± 69.8	0.574
HbA1c (%)	7.4 ± 1.6	7.4 ± 1.7	7.4 ± 1.5	0.931
Hemoglobin (g/dL)	11.9 ± 3.1	12.2 ± 1.7	11.7 ± 4.2	0.517
Phosphate (mg/dL)	3.5 ± 0.9	3.7 ± 0.9	3.4 ± 0.8	0.167
Median intact-PTH (pg/mL)	120.5 [66.9, 256.6]	133.55 [63.9, 269.2]	120.5 [70.6, 214.1]	0.887

Note: Values for categorical variables are given as number (percentage); values for continuous variables, as *mean ± standard deviation or median [interquartile range]*

*Abbreviations: ASA* Aspirin, *BMI* Body Mass Index, *DBP* Diastolic Blood Pressure, *FPG* Fasting Plasma Glucose, *GFR* Glomerular Filtration Rate, *HbA1c* Hemoglobin A1 C, *PTH* Parathyroid hormone, *RAAS* Renin Angiotensin Aldosterone System, *SBP* Systolic Blood Pressure, *UACR* Urine Albumin Creatinine Ratio

^a^A high cardiovascular risk was defined a 10-year cardiovascular disease ≥ 10% by Framingham Coronary Heart Disease Risk Score

### Comparisons between patients with rapid versus non-rapid renal progression

During the observational period, 37 patients (44.6%) had GFR decline ≥ 25% yearly and were classified as the rapid renal progression group. Mean baseline estimated GFR of subjects with rapid renal progression were significantly lower than those in the non-rapid renal progression group (*P* = 0.012). The patients with rapid renal progression had increased systolic and diastolic BP (Table 1). The prevalence of those with a history of cardiovascular event, the Framingham risk score or high cardiovascular risk were not different between rapid versus non-rapid progression.

**Fig. 1:**
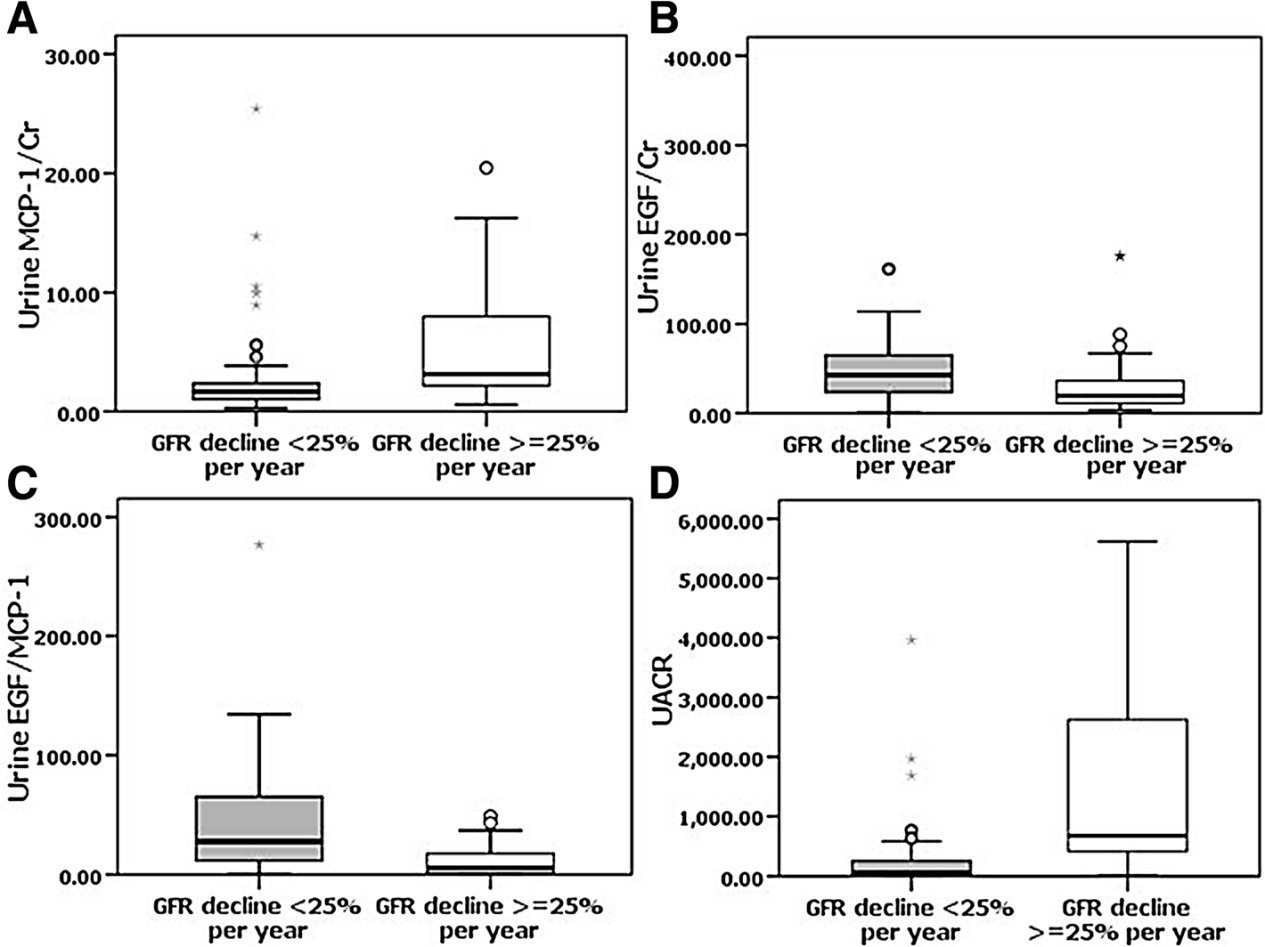
Urinary levels of renal biomarkers in the rapid and nonrapid GFR decline groups **a**. UMCP-1 (ng/mgCr), **b**. UEGF (ng/mgCr), **c** UEGF/MCP-1 (ng/ng) and **d**. UACR (mg/gCr) in T2DM patients classified in two groups according to GFR decline: rapid renal progression, non-rapid renal progression. There were significantly differences in UMCP-1, UEGF, UEGF/MCP-1 and UACR between the rapid renal progression and non-rapid renal progression group (*P* < 0.001)

UMCP-1 (Rapid: 3.12 [2.17, 7.97] vs. Non-rapid: 1.66 [1, 2.38] ng/mgCr (*P* < 0.001)) and UACR (Rapid: 673.4 [412.5 to 2627.6] vs. Non-rapid: 49.7 [19.9, 261.3] mg/gCr (*P* < 0.001)) were significantly higher in the rapid renal progression group compared to non-rapid renal progression group. In contrast, UEGF levels (Rapid: 19.5 [11.1, 36.3] vs. Non-Rapid: 42.8 [23.4, 65.1] ng/mgCr *(P* < 0.001)), and UEGF/MCP-1 ratios (Rapid: 5.4 [1.5, 17.5] vs. Non-rapid: 27.3 [11.5, 65.3] ng/ng (*P* < 0.001)), were significantly lower in the rapid renal progression group when compared with non-rapid renal progression group as shown in Fig. 1.

### Urine MCP-1 and EGF correlated with other renal injury markers

UMCP-1 showed an inverse correlation with estimated GFR (*R* = − 0.29, *P* = 0.008) and a strong positive correlation with albuminuria (*R* = 0.79, *P* < 0.001) at baseline. By contrast, UEGF showed a positive correlation with estimated GFR (*R* = 0.39, *P* < 0.001), but did not correlate with albuminuria (*R* = − 0.19, *P* = 0.078). UEGF/MCP-1 ratio showed a moderate correlation with estimated GFR (*R* = 0.63, *P* < 0.001) and a significant inverse correlation with albuminuria (*R* = − 0.34, *P* < 0.001) (Additional file 1: Table S1).

### Performance of the biomarkers to predict rapid renal progression

**Fig. 2:**
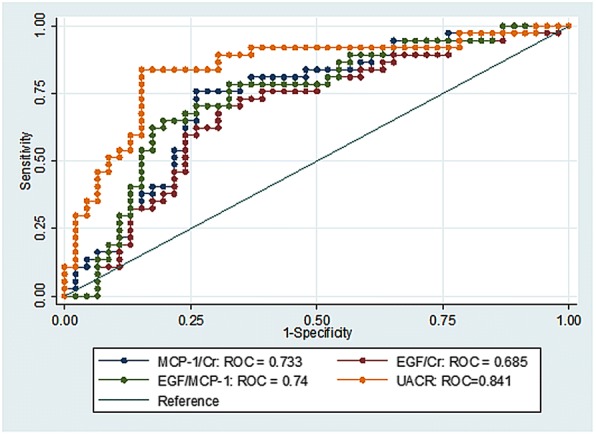
Graph ROC curves showing Area under the Curve (AUC) of each renal biomarker to predict rapid GFR decline

The ROC analysis showed an AUC (95%CI) for UMCP-1, UEGF, UEGF/MCP-1 ratio and UACR of 0.73 (0.62–0.84), 0.68 (0.57 to 0.80), 0.74 (0.63–0.85) and 0.84 (0.75–0.93), respectively as shown in Fig. 2. For UMCP-1, the best cut-off level was 2.1 ng/mgCr (sensitivity 75.7%, specificity 73.9%), UEGF was 29.9 ng/mgCr (sensitivity 70.3%, specificity 69.6%), UEGF/MCP-1 ratio was 9.2 ng/ng (sensitivity 64.9%, specificity 80.4%) and UACR was 330.9 mg/gCr (sensitivity 83.8%, specificity 84.8%). All renal biomarkers demonstrated intermediate performance to predict rapid renal progression in T2DM patients. When the levels of biomarkers were combined with UACR, The AUC (95%CI) for combined markers were: UACR+UMCP-1, 0.84 (0.75–0.93); UACR+UEGF, 0.83 (0.75–0.93); UACR +UEGF/MCP-1 0.82 (0.74–0.92). There was no improvement in the AUC compared to UACR alone.

**Fig. 3:**
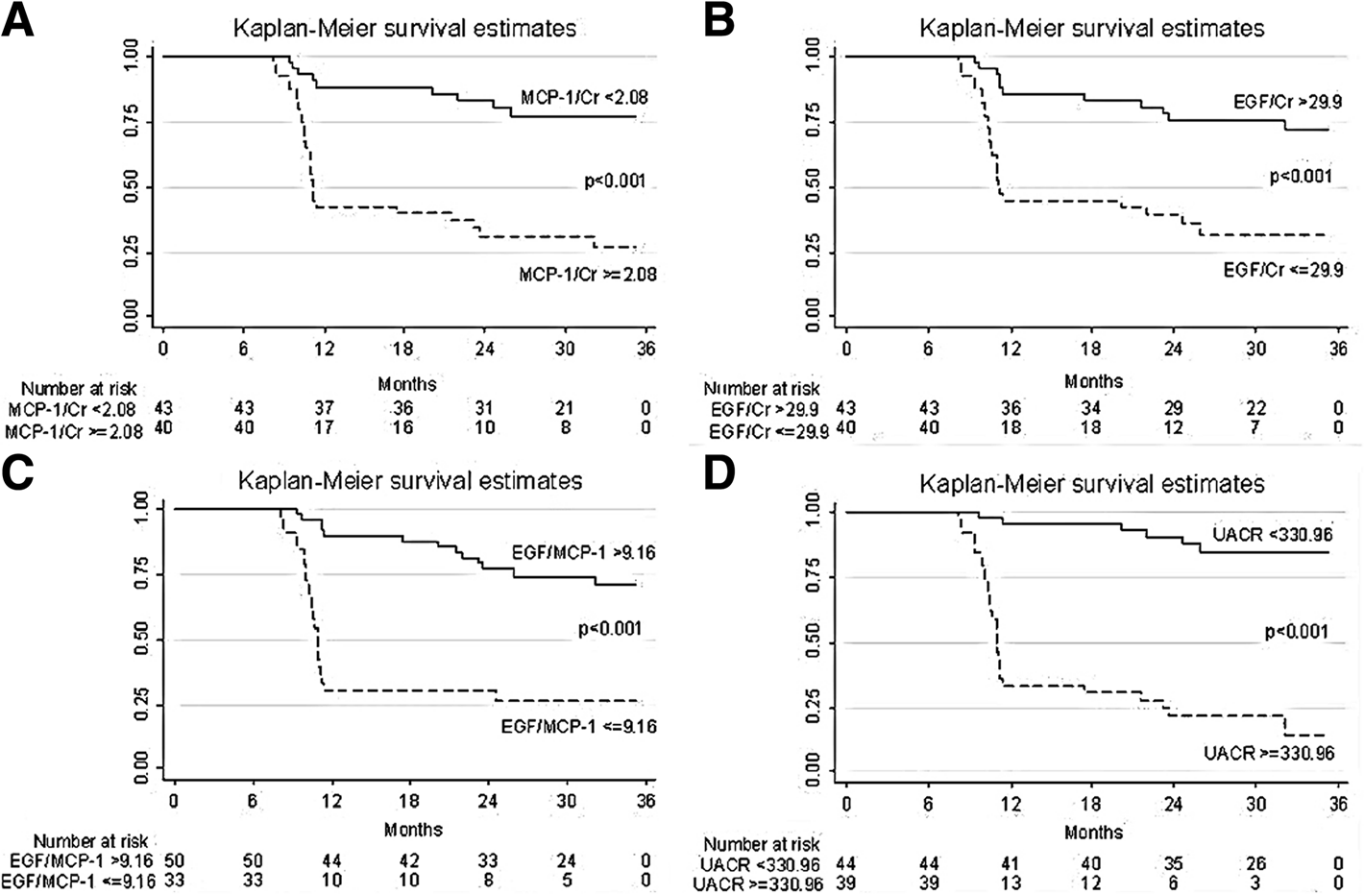
Kaplan-Meier survival curves of renal endpoint in patients with UMCP-1 (ng/mgCr), UEGF (ng/mgCr), UEGF/MCP-1 (ng/ng) and UACR (mg/gCr) above and below the optimal receiver operating characteristics cutoff level of each tubular biomarkers. **a**) UMCP-1 ≥ 2.08 ng/mgCr, **b**) UEGF ≤29.9 ng/mgCr, **c**) UEGF/MCP-1 ≤ 9.16 ng/ng, **d**) UACR ≥330.96 mg/gCr showed a significantly faster progression to endpoint (*p* < 0.001, log-rank test). *Abbreviations: UACR* urine albumin creatinine ratio, *UEGF* urine epidermal growth factor creatinine ratio, *UEGF/MCP-1* urine epidermal growth factor and monocyte chemoattractant protein-1 ratio, *UMCP-1* urine monocyte chemoattractant protein-1 creatinine ratio

Kaplan-Meier survival curves of *Rapid GFR decline* in patients with UMCP-1, UEGF, UEGF/MCP-1 ratio and UACR levels above and below the optimal cutoff defined by ROC analysis are presented in Fig. 3. Subjects with high UMCP-1 or UACR UEGF and low UEGF or UEGF/MCP-1 ratio values experienced a significantly faster evolution to endpoint (*P* < 0.001). SBP.

### Urine MCP-1 or EGF/MCP-1 ratio as independent predictors of rapid decline GFR

Univariate analysis of clinical and biomarkers showed that systolic blood pressure, diastolic blood pressure, GFR, UACR, UMCP-1, UEGF, and UEGF/MCP-1 ratio were associated with *Rapid GFR decline*. In multivariate regression analysis, systolic blood pressure, UMCP-1 in the model 1 and UEGF/MCP-1 ratio in the model 3 were independently associated with *Rapid GFR decline*, respectively (Table 2).

**Table 2:** Univariate and multivariate analysis of traditional and biomarkers for rapid GFR decline

Parameters	Univariate		Multivariate					
	HR (95% CI.)	*P* value	Model 1 HR (95% CI.)	*P* value	Model 2 HR (95% CI.)	*P* value	Model 3 HR (95% CI.)	*P* value
SBP (mmHg)	1.03 (1.02, 1.05)	< 0.001	1.03 (1.01, 1.06)	0.002	1.03 (1.01, 1.05)	0.015	1.02 (1, 1.04)	0.102
DBP (mmHg)	1.04 (1.01, 1.06)	0.006	1.02 (0.98, 1.05)	0.357	1.03 (0.99, 1.07)	0.119	1.04 (1, 1.08)	0.052
GFR (mL/min/1.73m^2^)	0.98 (0.97, 0.99)	0.003	0.99 (0.98, 1.01)	0.506	1 (0.98, 1.02)	0.852	1.01 (0.99, 1.03)	0.59
Age	0.99 (0.96, 1.02)	0.547	0.99 (0.96, 1.03)	0.776	1.02 (0.98, 1.06)	0.379	1.02 (0.99, 1.06)	0.211
Male	1.02 (0.52, 2.01)	0.947	1.54 (0.72, 3.31)	0.263	1.18 (0.54, 2.57)	0.677	1.07 (0.49, 2.35)	0.858
Use of RAAS blockers	0.59 (0.31, 1.14	0.115	0.64 (0.27, 1.5)	0.301	0.92 (0.4, 2.09)	0.834	0.8 (0.34, 1.9)	0.616
UACR (mg/gCr)	1.01 (1.01, 1.02)	< 0.001	1.01 (1.01, 1.02)	< 0.001	1.01 (1.01, 1.02)	< 0.001	1.01 (1.01, 1.02)	0.003
UMCP-1 (ng/mgCr)	1.03 (1.01, 1.05)	0.004	0.93 (0.89, 0.97)	0.001				
UEGF (ng/mgCr)	0.98 (0.97, 0.99)	0.011			0.99 (0.97, 1.01)	0.274		
UEGF/MCP-1 (ng/ng)	0.97 (0.95, 0.99)	0.001					0.97 (0.94, 0.99)	0.018

*Abbreviations: DBP* Diastolic Blood Pressure, *GFR* Glomerular Filtration Rate, *RAAS* Renin Angiotensin Aldosterone System, *SBP* Systolic Blood Pressure, *UACR* Urine Albumin Creatinine Ratio, *UMCP-1* urinary monocyte chemoattractant protein-1/creatinine, *UEGF* urinary epidermal growth factor/creatinine, *UEGF/MCP* urinary epidermal growth factor/urinary monocyte chemoattractant protein-1 ratio

### Subgroup analysis

#### Patients with high cardiovascular risk

Of all patients, 37 (44.6%) were classified as high cardiovascular risk. When patients with high cardiovascular risk were analyzed as a separate subgroup, none of the clinical or biomarker factors including UACR were significant predictors for rapid GFR decline by univariate analysis (*data not shown*).

#### Patients with low albuminuria

**Fig. 4:**
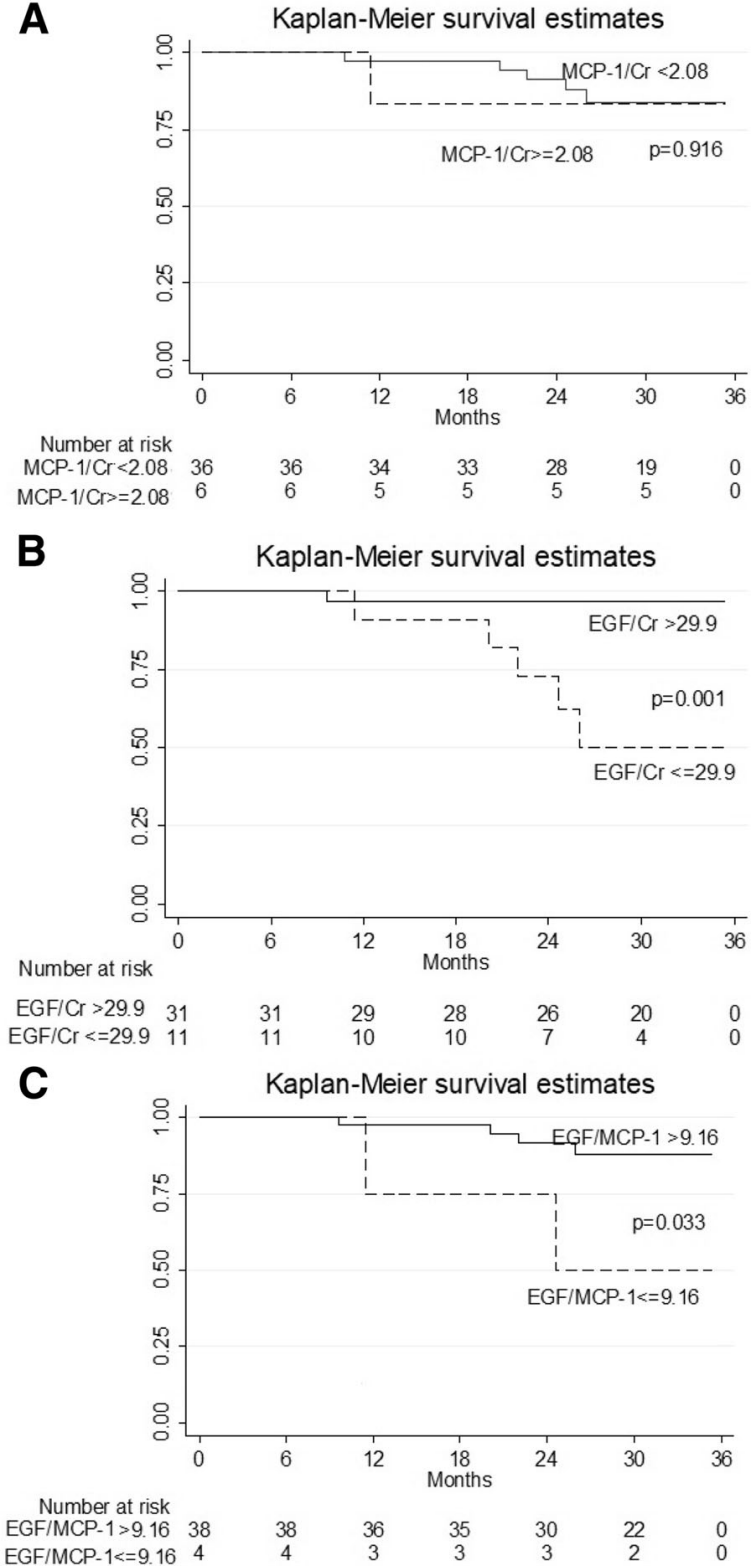
Kaplan-Meier survival curves of renal endpoint in low albuminuria patients (< 330.9 mg/gCr of median UACR) with **a**) UMCP-1 (ng/mgCr), **b**) UEGF (ng/mgCr), and **c**) UEGF/MCP-1 (ng/ng) above and below the optimal receiver operating characteristics cutoff level of each tubular biomarkers. Only UEGF ≤30 ng/mgCr, and UEGF/MCP-1 ≤ 9.2 ng/ng showed a significantly faster progression to endpoint (*p* < 0.001, log-rank test)

We further evaluated the sub-group of patients with low albuminuria (UACR< 330.9 mg/gCr of median UACR). Figure 4 showed that among patients with low albuminuria, patients with either high EGF or EGF/MCP-1 had significantly faster progression to end-point by univariate analysis.

## Discussion

The ability to identify which DKD patients will develop rapid decline in kidney function is essential and currently available clinical tests cannot predict renal outcomes accurately. The present study indicate that MCP-1, EGF, and the EGF/MCP-1 ratio represent potential biomarkers of rapid progression in DKD. UMCP-1, and UEGF/MCP-1 ratio showed significant predictive power after multivariate analysis with conventional factors (blood pressure, GFR and UACR). This might suggest that these biomarkers would not be simple surrogate indexes of baseline estimated GFR, but potential markers on their own, predicting DKD progression beyond the conventional risk factors.

The recruitment of inflammatory cells into renal tissue has a pivotal role in the progression of various renal diseases by promoting a microenvironment that amplifies tissue injury and fibrosis [20, 21]. MCP-1-mediated macrophage accumulation and activation are critical events in the development of diabetic renal injury in animal models [20, 21]. MCP-1 protein and mRNA were detected in cortical tubuli, and infiltrating mononuclear cells in the kidneys of patients with DKD [10], Urinary MCP-1 levels correlated with the severity of both the tubulointerstitial and glomerular lesions. Other studies have also shown correlations between urinary MCP-1 with baseline proteinuria and renal function in DKD [10, 22, 23]. In line with previous studies, we also found that UMCP-1 strongly correlated with the level of albuminuria. In addition, we also found that high UMCP-1 was a predictor of rapid GFR loss in DKD. The relationship between urinary MCP-1 levels and subsequent GFR decline had been observed in earlier studies. In a small study, urine MCP-1 levels was found to correlate with the rate of renal function decline in DKD patients over a 6 year period [24], but the authors did not adjust for conventional risk factors in the study. Recently, urinary MCP-1 was found to be independently associated with the rate of GFR decline in a Canadian cohort with advanced stage 3 to 4 DKD [25]. Our study extended previous observations by showing that high UMCP-1 was predictive of rapid renal function loss across a broad spectrum of kidney function that was independent of conventional factors in Asian patients with stage 1 to 5 DKD.

EGF plays important role in restoring barrier function in the healing phase of renal injury and is also a critical tubular cell survival factor [26, 27]. A role for EGF in predicting outcome in chronic kidney diseases is being increasingly recognized, but there is limited data in DKD. Urinary EGF levels have been shown to correlate with the severity of tubulointerstitial fibrosis in primary glomerulonephritis [13], and low urinary EGF has been shown to be a risk predictor of kidney progression in non-diabetic kidney diseases [28]. More recently, urine EGF was identified as a marker for DKD by urine peptidomic profiling of a diabetic rodent model [29], and reduced urinary EGF levels had been reported in patients with DKD [30, 31]. In this study, we demonstrated the predictive role of low UEGF for detecting rapid kidney decline across a broad spectrum of GFR and albuminuria including a subgroup of patients with low albuminuria (mostly in the microalbuminuria range). Previously, the predictive role of urinary EGF has been investigated in diabetic patients with preserved GFR and normoalbuminuria [29]. The investigators found that low urinary EGF was associated with rapid decline in renal function and predicted incident CKD independent of standard risk factors. DKD is associated with many alterations in the structure of glomeruli, tubulointerstitial and vascular compartments [32]. In line with previous studies, we showed that UEGF correlated with GFR, but not with albuminuria consistent with a predominant role of UEGF as a marker of renal tubulointerstitial involvement than of glomerular damage [13, 33]. However, unlike the previous study of patients with preserved GFR and normoalbuminuria [29], the utility of urine EGF to predict rapid decline in GFR was not apparent after adjustments for albuminuria in our study. Overall, this data is consistent with the hypothesis that EGF may be important in the pathogenesis of DKD and low UEGF may be a useful marker for progression especially in patients with normo- or low albuminuria. It is possible that the benefit of UEGF as a biomarker of rapid progression over traditional markers may be diminished in the context of more advanced DKD or marked proteinuria.

DKD is typically characterized by a high prevalence of cardiovascular risk factors and increased risk for cardiovascular events [2]. Macrovascular disease has been proposed as a mechanism for decreased GFR in some DKD patients [3, 4]. Biomarkers that can identify either cardiovascular or renal outcomes in patients with high cardiovascular risk would be especially useful as interventions may have the greatest benefit in this group [2]. We explored the role of the biomarkers specifically in a subgroup with high cardiovascular risk defined as > 10% probability for developing cardiovascular event at 10 years based on the Framingham risk score. Although the proportion with high risk was quite high (44.6%), only 3.7% actually had already had CV events. Thus most patients may not have extensive macrovascular disease at baseline. The proportion with high cardiovascular risk or the Framingham risk score at baseline did not differ between those who developed rapid GFR decline compared to those who did not suggesting that high CV risk might not be a major factor for GFR decline in our study population. None of the clinical parameters or biomarkers were predictive of rapid GFR decline in the high cardiovascular risk subgroup, which probably reflected the small number of patients in the subgroup. Larger studies are necessary to allow firm conclusions on the role of these biomarkers in high cardiovascular risk patients.

Albuminuria is commonly used to assess kidney disease progression among patients with DKD, but some studies have shown that this marker may have insufficient ability to predict kidney disease end points on its own [19]. On this basis, exploring novel biomarkers that can more reliably identify individuals at risk of experiencing adverse renal outcomes is warranted. Inflammatory cytokines and growth factors are logical candidates for biomarkers of progressive DKD as they have been involved in the pathogenesis of renal fibrosis and are easily measurable in the urine. In our study, albuminuria was still the strongest predictor for rapid GFR decline. The benefit of adding additional biomarkers varied depending on the statistical method used. The C-statistics test showed that UACR had a fairly good discrimination for rapid GFR decline. The addition of other biomarkers did not improve the area under the curve. However, the C-statistics test may be insensitive when adding a new predictor to a model and may occasionally produce incorrect estimates [34]. This phenomenon is particularly perceptible when the baseline model includes strong predictors especially in the context of low numbers of patients such as in our study. On the other hand, by using the multivariate model, we showed that MCP-1 or EGF/MCP-1 were independent predictors of rapid GFR decline. Together with findings from previous studies, our results suggest that urine MCP-1 could be a promising biomarker for rapid GFR decline in DKD across a broad range of GFR, but additional studies are necessary to confirm the added benefit to conventional factors including UACR. Findings of this study has several implications. Urine MCP-1 might be utilized to identify high risk patients for inclusion into clinical trials or for more aggressive intervention. The findings also provide additional support for the role of MCP-1 in the pathogenesis of human DKD progression. This is especially important as specific inhibitors of MCP-1 are now available for clinical studies with the potential to impact proteinuria [35]. It would be of interest to see if high urinary MCP-1 could identify those who would mostly likely to respond to MCP-1 blockade. To our knowledge, this is the first study to evaluate the role of EGF in addition to MCP-1 as a ratio in DKD. Earlier studies have shown that combinations of EGF with MCP-1 in the form of EGF/MCP-1 ratio was a better predictor of eGFR decline in non-diabetic kidney disease compared to either biomarker alone [14]. In this study, although high EGF/MCP-1 was an independent of rapid loss of GFR in DKD, the overall performance of the ratio was comparable to UMCP-1 alone. Therefore, our study does not support the benefit of routine EGF measurement in addition to MCP-1 given the additional costs. EGF or EGF/MCP-1 may be useful in the DKD subgroup with normo- or low grade albuminuria and decreased GFR. This subgroup may constitute a larger proportion of DKD than previously recognized [2, 4] but larger studies are necessary to evaluate the cost-benefit of EGF measurement to predict adverse renal outcomes in this group.

This study has several limitations. This is a single center study involving small numbers of subjects with a short follow-up period of 24 months. Urine biomarkers were measured in a cohort of high risk of DKD patients who might have had a more rapid decline in renal function in a limited time of follow-up. The inclusion of higher risk patients and single-center nature of the study design may have an impact on its external validity to a broader group of T2DM patients. It remains to be seen if urinary MCP-1 and or EGF really predict future loss of kidney function in more or less stable, comparable patients or if these values are “just” independently associated with more rapid loss of kidney function. In keeping with standard clinical practice, we did not perform kidney biopsies routinely in patients with DKD in the absence of clinical features suggestive of other kidney diseases [3]. The prevalence of non-diabetic kidney diseases in patients with diabetes reported in the literature is highly variable, but about 10% of unselected patients with early DKD and albuminuria may have non-DKD as an underlying kidney disease [3]. A number of our patients had low albuminuria. However, a lack of albuminuria may not necessarily preclude structural DKD and almost all histopathologic classes of DKD may be present in such patients [36]. However, given that our inclusion criteria of poorly controlled hypertension or longer duration of diabetes, other diagnoses such as hypertensive nephropathy or other glomerular diseases could not be excluded. Finally, given the limited time, our study did not collect data of major renal outcomes such as ESRD.

## Conclusions

This study suggests that urinary MCP-1, EGF and EGF/MCP-1 ratio may be promising markers of renal progression among T2DM patients. High urinary MCP-1 may be an independent predictor for rapid GFR decline after adjustments for conventional markers. Further prospective studies in larger populations with longer follow-up are warranted to fully evaluate the roles of urine MCP-1 or EGF/MCP-1 in disease management or as a guide for clinical trial entry in DKD.

## Additional file

Additional file 1: **Table S1.** Correlation of baseline urine MCP-1, EGF and EGF/MCP-1 ratio with other markers of renal injury. Relationship between biomarkers and clinical and laboratory parameters at baseline. (DOCX 19 kb)

## Abbreviations

AUC: Area under the curves
CKD-EPI: Chronic Kidney Disease Epidemiology Collaboration
DKD: Diabetic kidney disease
EGF: Epidermal growth factor
ESRD: End stage renal disease
GFR: Glomerular filtration rate
MCP-1: Monocyte chemoattractant protein-1
ROC: Receiver operating characteristic
T2DM: Type 2 diabetes mellitus
UACR: Urine albumin creatinine ratio
UEGF: Urine epidermal growth factor creatinine ratio
UEGF/MCP-1: Urine epidermal growth factor epidermal growth factor ratio
UMCP-1: Urine monocyte chemoattractant protein-1 creatinine ratio
