# Supplementary material for: Urinary epidermal growth factor, monocyte chemoattractant protein-1 or their ratio as predictors for rapid loss of renal function in type 2 diabetic patients with diabetic kidney disease
Source: BMC Nephrol. 2018 Sep 21;19:246. doi: 10.1186/s12882-018-1043-x (PMC6150979; doi:10.1186/s12882-018-1043-x)
Supplement: Supplementary file 1 — Table S1. Correlation of baseline urine MCP-1, EGF and EGF/MCP-1 ratio with other markers of renal injury. Relationship between biomarkers and clinical and laboratory parameters at baseline. (DOCX 19 kb) [file 12882_2018_1043_MOESM1_ESM.docx]

**Table S1. Correlation of baseline urine MCP-1, EGF and EGF/MCP-1 ratio with other markers of renal injury**

| **Parameters** | **UMCP-1**  **(ng/mgCr)** | | **UEGF**  **(ng/mgCr)** | | **UEGF/MCP**  **(ng/ng)** | |
| --- | --- | --- | --- | --- | --- | --- |
|  | r | p | r | p | r | p |
| Age (year) | -0.25 | 0.021 | -0.02 | 0.869 | -0.06 | 0.607 |
| Male (%) | 0.03 | 0.779 | 0.01 | 0.993 | 0.01 | 0.974 |
| Duration of DM (years) | -0.11 | 0.383 | 0.01 | 0.915 | 0.09 | 0.452 |
| SBP (mmHg) | 0.27 | 0.014 | 0.04 | 0.708 | -0.26 | 0.017 |
| DBP (mmHg) | 0.20 | 0.065 | 0.19 | 0.088 | 0.08 | 0.487 |
| BMI (kg/m^2^) | 0.04 | 0.772 | 0.01 | 0.935 | -0.12 | 0.353 |
| BUN (mg/dL) | 0.14 | 0.245 | -0.24 | 0.038 | -0.42 | <0.001 |
| Serum creatinine (mg/dL) | 0.27 | 0.013 | -0.30 | 0.006 | -0.45 | <0.001 |
| GFR (mL/min/1.73m^2^) | -0.29 | 0.008 | 0.39 | <0.001 | 0.63 | <0.001 |
| UACR (mg/g) | 0.79 | <0.001 | -0.19 | 0.078 | -0.34 | <0.001 |
| FPG (mg/dL) | -0.03 | 0.801 | -0.07 | 0.531 | -0.10 | 0.348 |
| HbA1c (%) | -0.04 | 0.755 | 0.02 | 0.865 | -0.08 | 0.511 |
| Hemoglobin (g/dL) | -0.19 | 0.127 | 0.16 | 0.204 | 0.13 | 0.303 |
| Phosphate (mg/dL) | -0.15 | 0.272 | -0.09 | 0.485 | -0.08 | 0.570 |
| Intact-PTH (pg/mL) | -0.02 | 0.917 | -0.32 | 0.152 | -0.34 | 0.136 |
| Cholesterol (mg/dL) | 0.06 | 0.618 | -0.09 | 0.484 | -0.09 | 0.472 |
| LDL-cholesterol (mg/dL) | 0.22 | 0.055 | -0.12 | 0.302 | -0.12 | 0.292 |

Abbreviations: ASA, Aspirin; BMI, Body Mass Index; DBP, Diastolic Blood Pressure; FPG, Fasting Plasma Glucose; GFR, Glomerular Filtration Rate; HbA1c, Hemoglobin A1 C; PTH, Parathyroid hormone; RAAS, Renin Angiotensin Aldosterone System; SBP, Systolic Blood Pressure; UACR, Urine Albumin Creatinine Ratio; UMCP-1, urinary monocyte chemoattractant protein-1/creatinine; UEGF, urinary epidermal growth factor/creatinine; UEGF/MCP, urinary epidermal growth factor/ urinary monocyte chemoattractant protein-1

Ratio
